# Preoperative Risk Score for Predicting Delayed Wound Healing After Plate Fixation in Patients With Closed Distal Fibular Fractures

**DOI:** 10.7759/cureus.110995

**Published:** 2026-06-16

**Authors:** Masaya Mizutani, Toshiaki Kotani, Shun Okuwaki, Shuhei Ohyama, Shuhei Iwata, Seiji Kimura, Kazuhide Inage, Sumihisa Orita, Shohei Minami, Seiji Ohtori

**Affiliations:** 1 Department of Orthopedic Surgery, Seirei Sakura Citizen Hospital, Sakura, JPN; 2 Department of Orthopedic Surgery, Tsukuba University, Tsukuba, JPN; 3 Department of Orthopedic Surgery, Chiba University, Chiba, JPN; 4 Department of Orthopedic Surgery, Chiba University Hospital, Chiba, JPN; 5 Department of Orthopedic Surgery, Graduate School of Medicine, Chiba University, Chiba, JPN

**Keywords:** delayed wound healing, distal fibular fracture, open reduction and internal fixation, postoperative wound complication, preoperative risk scoring system

## Abstract

Background: Delayed wound healing after open reduction and internal fixation (ORIF) of distal fibular fractures can impair recovery and increase infection risk. The purpose of this study was to develop a simple preoperative scoring system to predict delayed wound healing after plate fixation in patients with closed distal fibular fractures.

Methods: This retrospective cohort study reviewed 84 patients who underwent plate fixation for closed distal fibular fractures between 2018 and 2024. Delayed wound healing was defined as a surgical wound rated as Grade 2 or higher according to the Southampton Wound Assessment Scale postoperatively. The following preoperative variables were collected, binarized, and entered into a logistic regression model to develop a weighted risk score: age, diabetes mellitus status, fracture characteristics, and planned number of plates.

Results: Delayed wound healing occurred in 30 patients. Multivariate analysis identified age ≥70 years, complex fracture type, and use of ≥2 plates as independent predictors of delayed wound healing. Presence of diabetes mellitus was retained for clinical relevance. Each predictor was weighted as follows: age and diabetes, one point each; fracture type and planned use of ≥2 plates, two points each. These scores demonstrated good predictive performance.

Conclusion: We developed a simple preoperative scoring system for predicting delayed wound healing following ORIF for closed distal fibular fractures using four clinical variables, supporting early risk stratification. This model may aid in identifying high-risk patients preoperatively, allowing for informed surgical planning and individualized perioperative care to minimize wound complications.

## Introduction

Postoperative wound complications are a common concern following open reduction and internal fixation (ORIF) of distal fibular fractures, occurring in 5%-40% of patients. Delayed wound healing, wound dehiscence, and superficial infection have been reported in up to 19% of cases [1,2]. Delayed wound healing and surgical site infection (SSI) are related but distinct postoperative wound complications. SSI represents an established infectious complication, whereas delayed wound healing can be identified earlier in the postoperative course and may precede subsequent infection or prolonged wound management [3]. Therefore, delayed wound healing was selected as the primary endpoint in this study because it reflects an early and clinically relevant wound-related problem after ankle fracture surgery.

Previous studies have identified several risk factors for wound complications, including diabetes mellitus, smoking, advanced age, male sex, and peripheral vascular disease [1,4-6]. Injury- and procedure-related factors, such as fracture-dislocation, trimalleolar involvement, external fixation, and longer surgical time, have also been implicated [4,6-8]. Despite this knowledge, no validated scoring system exists for predicting delayed wound healing specifically in patients with closed distal fibular fractures undergoing ORIF. Existing risk scoring systems mainly focus on general orthopedic trauma-related SSIs or heterogeneous orthopedic procedures and do not specifically target delayed wound healing in this patient population [9].

The aim of this study was to develop a simple preoperative scoring system to predict delayed wound healing after plate fixation in patients with closed distal fibular fractures. This scoring system was intended to support preoperative risk stratification, patient counseling, surgical planning, and early postoperative wound surveillance using readily accessible clinical variables.

## Materials and methods

Study design and patient selection

This retrospective, single-center cohort study was conducted between 2018 and 2024. This study enrolled patients who were diagnosed with a distal fibular fracture in conjunction with an ankle fracture and underwent surgery between April 2018 and April 2024. Patients were considered eligible if they underwent primary ankle fracture surgery with plate fixation and had complete perioperative medical records available. In contrast, patients were excluded if they presented with open fractures, required revision surgery, sustained polytrauma, or had incomplete follow-up data within 30 days postoperatively.

Data collection and variables

Data on preoperative variables were collected from the institutional electronic medical record system, including demographic data (age, sex, weight, height, and body mass index (BMI)), comorbidities (diabetes mellitus status and smoking habit), fracture characteristics determined using radiography and computed tomography, and operation strategy, including the planned use of ≥2 plates based on preoperative radiographs and computed tomography. Fracture characteristics included dislocation, medial malleolar fracture, posterior malleolar fracture, and pilon-type fracture. In this study, pilon-type fractures were defined as distal tibial intra-articular fractures, mainly posterior malleolar fractures with an axial-loading component, rather than classic high-energy, severely comminuted tibial plafond fractures. All included cases were closed injuries with an associated distal fibular fracture requiring plate fixation.

Fracture characteristics were assessed using preoperative radiographs and computed tomography by orthopedic surgeons. For the scoring model, fracture complexity was defined as medial or posterior malleolar involvement or dislocation.

Surgical procedure and perioperative management

Orthopedic surgeons performed surgical procedures in accordance with the standard practice of our institution. Distal fibular fractures were treated with plate fixation through a lateral or posterolateral approach. The operative approach, implant selection, and planned use of ≥2 plates were determined preoperatively based on fracture morphology, soft-tissue condition, and radiographic and computed tomography findings. Additional fixation for medial or posterior malleolar fractures was performed when considered necessary based on fracture morphology and surgical planning.

Wound closure, perioperative antibiotic prophylaxis, and postoperative wound care were performed according to institutional protocols. Postoperative wound management included routine wound inspection and dressing changes, with additional wound care, including debridement, performed when clinically indicated.

Definition of delayed wound healing

Based on previous studies [10-12], patients were defined as having delayed wound healing if they were classified as Grade 2 or higher on the Southampton Wound Assessment Scale, originally described by Bailey et al. [11], on postoperative day seven and continued to receive wound care--such as dressing changes, wound inspection, and debridement--as documented in electronic medical records and confirmed by the attending surgeon. Patients with early wound closure and no signs of infection or wound-related complaints, corresponding to Grade 0 or 1, were classified as having normal healing.

Wound status was evaluated by the attending surgeon during routine postoperative care. The diagnosis of delayed wound healing was based on the Southampton Wound Assessment Scale, electronic medical record documentation, and confirmation by the attending surgeon.

Statistical analysis and scoring system development

All statistical analyses were conducted using SPSS Statistics v.25 (IBM Corp., Armonk, NY, USA), with statistical significance set at a p-value <0.05. Univariate analyses were performed to identify potential predictors of delayed wound healing using the chi-square test for categorical variables and the t-test for continuous variables. Continuous variables are expressed as the mean and standard deviation, whereas categorical variables are expressed as numbers and percentages.

To construct a clinically applicable predictive model, we selected four clinically relevant variables and transformed them into binary form (0 = absent; 1 = present) based on the following standard clinical thresholds: age (<70 vs. ≥70 years), diabetes mellitus status (absent vs. present), fracture type (simple vs. medial/posterior malleolus involvement or dislocation), and planned number of plates (1 vs. ≥2 plates). These variables were chosen based on the available literature, clinical plausibility, preoperative availability, and the need to avoid overfitting given the limited number of delayed wound healing events. We then conducted a univariate analysis to compare these variables between the control (n = 54) and delayed wound healing (n = 30) groups. The variables were also included in the multivariate logistic regression analysis.

A risk score was then constructed based on the regression coefficients (β values) obtained from the final model. Each variable was assigned a weighted score proportional to its coefficient, and the total score was calculated as the sum of these values.

To evaluate the internal validity of the predictive model, we performed bootstrapping with 1,000 resamples. In each iteration, a logistic regression model was trained on a bootstrap sample drawn with replacement, and the area under the receiver operating characteristic curve (AUC-ROC) was calculated using the original dataset as the test set. The mean AUC and 95% confidence interval (CI) were derived from the bootstrap distribution, and the discriminatory performance of the scoring system was evaluated using the AUC-ROC. Moreover, to evaluate potential multicollinearity among the predictor variables, we calculated the variance inflation factor (VIF) for the following variables: planned use of ≥2 plates and fracture type.

This retrospective study was conducted using data obtained for clinical purposes. Owing to the retrospective design of the study, the need for obtaining informed consent was waived, and the patients were given the option to withdraw from the study using the opt-out approach, which was available on our website.

## Results

In total, 84 patients who underwent plate fixation for distal fibular fractures were included in this study, of whom 30 (35.7%) experienced delayed wound healing, defined as requiring continued wound care beyond postoperative day seven. The remaining 54 patients (64.3%) comprised the normal healing (control) group.

Table 1 presents a comparison of the demographic and clinical characteristics between patients with and those without delayed wound healing. Notably, the mean age and prevalence of diabetes mellitus were significantly higher in the delayed healing group than in the control group (61.7 ± 19.7 vs. 50.7 ± 20.6 years, t = 2.37, p = 0.02; and 30.0% vs. 9.1%, χ² = 4.71, p = 0.03, respectively). Several fracture and surgical factors were also significantly associated with delayed wound healing, including pilon-type fracture (33.3% vs. 1.8%, χ² > 10.83, p < 0.001), fracture-dislocation (50.0% vs. 14.5%, χ² > 10.83, p < 0.001), concomitant medial malleolus fracture (76.7% vs. 30.9%, χ² > 10.83, p < 0.001), concomitant posterior malleolus fracture (53.3% vs. 29.1%, χ² = 4.22, p = 0.04), use of external fixation (43.3% vs. 7.3%, χ² > 10.83, p < 0.001), and planned use of ≥2 plates (33.3% vs. 1.8%, χ² > 10.83, p < 0.001). In contrast, no significant differences in height, weight, BMI, sex, or smoking history were observed between the groups.

**Table 1:** Comparison of demographic and clinical characteristics between patients with and those without delayed wound healing. Data are presented as mean ± standard deviation or n (%). ^†^Pilon fracture includes intra-articular distal tibial fractures classified as AO/OTA 43B/C. BMI: body mass index; *p <0.05.

Characteristics	Control group (n = 54)	Delayed wound healing group (n = 30)	Test statistic	p-value
Age, years	50.7 ± 20.6	61.7 ± 19.7	t = 2.37	0.02*
Height, cm	165.1 ± 10.8	162.8 ± 9.8	t = 0.98	0.33
Weight, kg	69.5 ± 16.2	70.4 ± 15.2	t = 0.28	0.78
BMI, kg/m²	25.3 ± 4.2	26.5 ± 4.6	t = 1.18	0.24
Sex (male)	22 (40.0)	16 (53.3)	χ² = 1.27	0.26
Diabetes mellitus	5 (9.1)	9 (30.0)	χ² = 4.71	0.03*
Smoking history	5 (9.1)	5 (16.7)	χ² = 1.03	0.31
Pilon-type fracture†	1 (1.8)	10 (33.3)	χ² > 10.83	<0.001*
Fracture-dislocation	8 (14.5)	15 (50.0)	χ² > 10.83	<0.001*
Medial malleolus fracture	17 (30.9)	23 (76.7)	χ² > 10.83	<0.001*
Posterior malleolus fracture	16 (29.1)	16 (53.3)	χ² = 4.22	0.04*
External fixation	4 (7.3)	13 (43.3)	χ² > 10.83	<0.001*
Planned use of ≥2 plates	1 (1.8)	10 (33.3)	χ² > 10.83	<0.001*

Table 2 presents the univariate analysis results of the identified predictive clinical variables between the control (n = 54) and delayed wound healing (n = 30) groups. The delayed wound healing group had significantly higher proportions of patients aged ≥70 years (43.3% vs. 14.8%, χ² = 7.26, p = 0.007), patients with diabetes mellitus (30.0% vs. 9.3%, χ² = 4.76, p = 0.029), complex fracture types (86.7% vs. 42.6%, χ² > 10.83, p < 0.001), and planned use of ≥2 plates (33.3% vs. 1.8%, χ² > 10.83, p < 0.001) than the control group. Multivariate logistic regression analysis of these four variables showed that age ≥70 years (OR: 3.1; 95% CI: 1.12-8.74; p = 0.03), complex fracture type (OR: 7.9; 95% CI: 1.87-32.98; p = 0.005), and planned use of ≥2 plates (OR: 9.0; 95% CI: 2.66-30.75; p = 0.001) remained significant independent predictors of delayed wound healing (Table 3). Although diabetes mellitus status did not reach statistical significance in the multivariate model (OR: 2.5; 95% CI: 0.96-6.52; p = 0.06), it was retained in the final scoring system owing to its clinical importance and borderline statistical significance.

**Table 2:** Frequency of selected predictive variables in the control and delayed healing groups. Data are presented as n (%). ^†^Simple vs. medial/posterior malleolus involvement or dislocation. *p <0.05.

Predictive variable	Control group (n = 54)	Delayed wound healing group (n = 30)	Test statistic	p-value
Age ≥70 years	8 (14.8)	13 (43.3)	χ² = 7.26	0.007*
Diabetes mellitus	5 (9.3)	9 (30.0)	χ² = 4.76	0.029*
Fracture type†	23 (42.6)	26 (86.7)	χ² > 10.83	<0.001*
Planned use of ≥2 plates	1 (1.8)	10 (33.3)	χ² > 10.83	<0.001*

**Table 3:** Logistic regression coefficients and assigned scores for the predictive model. ^†^Simple vs. medial/posterior malleolus involvement or dislocation. CI: confidence interval; *p <0.05.

Variable	Coefficient (β)	Odds ratio	95% CI	p-value
Age ≥70 years	1.1	3.1	1.12–8.74	0.03*
Diabetes mellitus	0.9	2.5	0.96–6.52	0.06
Fracture type†	2.1	7.9	1.87–32.98	0.005*
Planned use of ≥2 plates	2.2	9.0	2.66–30.75	0.001*

**Figure 1:**
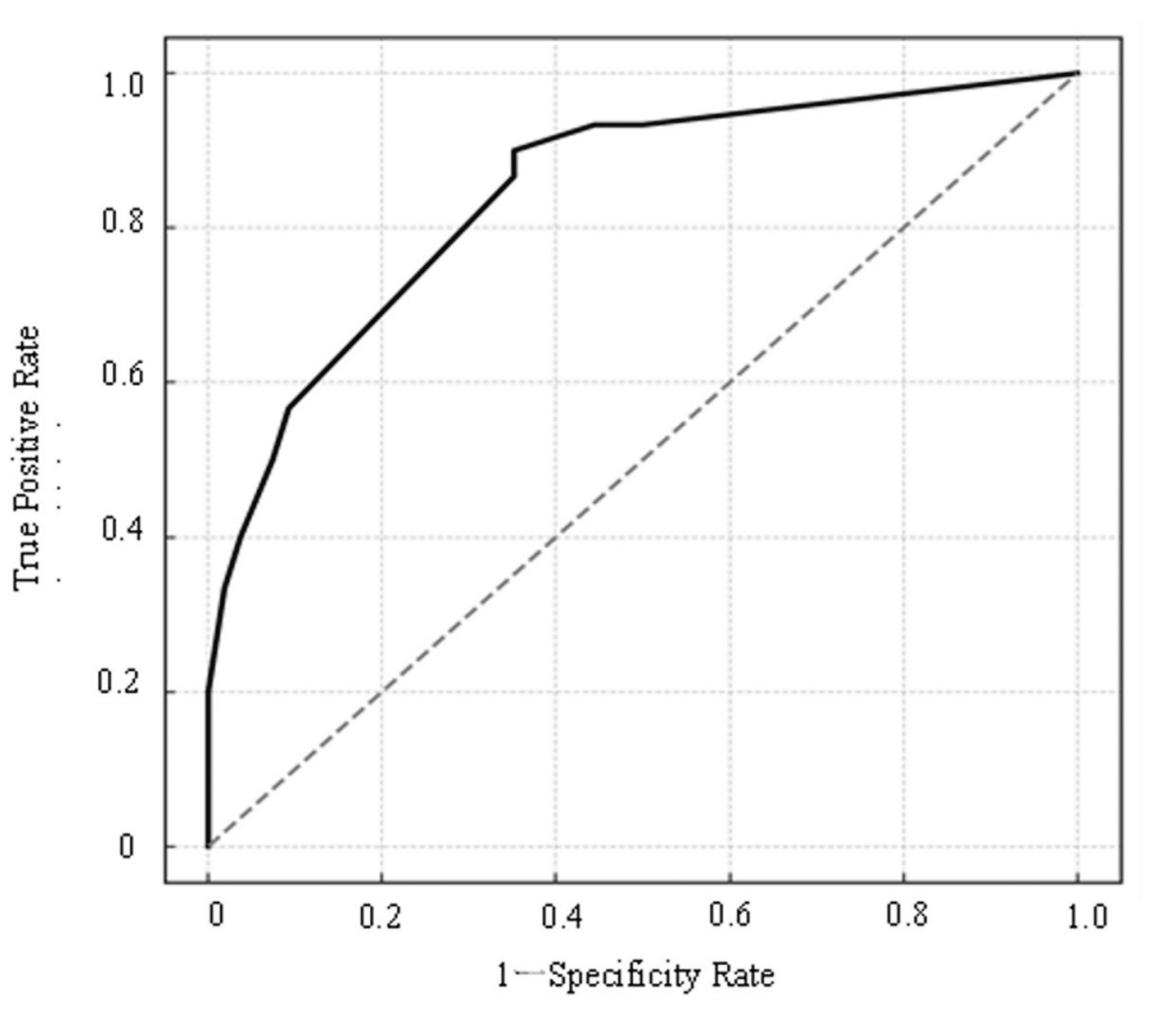
Receiver operating characteristic curve of the scoring model.

Upon construction of a weighted risk score by rounding the logistic regression coefficients of each variable to the nearest positive integer for practical use, the final score components were as follows: age ≥70 years, one point; diabetes mellitus, one point; fracture type, two points; and planned use of ≥2 plates, two points. Therefore, the total risk score ranged from 0 to 6. ROC curve analysis of this scoring system demonstrated good predictive performance, with an AUC of 0.85 (Figure 1). Stratification of patients into high- and low-risk groups using a cut-off score of 2 showed that the incidence of delayed wound healing increased proportionally with higher scores. Table 4 shows that higher cut-off scores improved precision in detecting delayed wound healing. At a cut-off of two points, 51 patients were classified as high risk, and the positive predictive value (PPV) was 54.9%. At a cut-off score of 3, 23 patients were classified as high risk, of whom 17 developed delayed wound healing, corresponding to a PPV of 73.9%. At a cut-off score of 4, 14 patients were classified as high risk, of whom 12 developed delayed wound healing, corresponding to a PPV of 85.7%.

**Table 4:** Positive predictive value and number of high-risk patients at different cut-off scores.

Cut-off score	Positive predictive value (%)	Number of high-risk patients	Sensitivity	Specificity
2	54.9%	51	0.93	0.58
3	73.9%	17	0.57	0.89
4	85.7%	14	0.40	0.96

In the bootstrap validation, the average AUC was 0.84 (95% CI: 0.81-0.85), indicating good model discrimination. Moreover, the VIF values for both the planned use of ≥2 plates and fracture type were 1.15, indicating no evidence of significant multicollinearity.

## Discussion

In the present study, a practical preoperative scoring system was developed for the prediction of delayed wound healing after ORIF for closed distal fibular fractures. We identified four key risk factors: age ≥70 years, presence of diabetes mellitus, complex fracture type, and planned use of ≥2 plates. A weighted score was constructed based on these factors and demonstrated promising discriminatory performance (AUC = 0.85). However, because the model was derived from a relatively small single-center cohort and internally validated only, this scoring system should be regarded as a preliminary predictive model until external validation is performed. This tool addresses the need for specific preoperative risk stratification methods for this common injury.

In the present study, we identified older age, complex fracture patterns, and the planned use of multiple plates as significant risk factors for delayed wound healing. These findings are consistent with those of previous studies, indicating that patient comorbidities, such as advanced age, diabetes mellitus, and fracture complexity, contribute to impaired wound outcomes [13-16]. However, to the best of our knowledge, no previous studies have identified the number of fixation plates as an independent predictor of delayed wound healing after distal fibular fracture fixation, although previous studies have reported higher risks for surgical site complications when multiple plates are used for the fixation of tibial plateau fractures [17-19]. This may be explained by the anatomical complexity and soft tissue vulnerability around the ankle, which could contribute to the increased risk associated with the use of multiple plates, as demonstrated in the present study.

Notably, in the multicollinearity analysis, although complex fracture types and the planned use of ≥2 plates were associated, they showed limited multicollinearity (VIF = 1.15). Therefore, as fracture type represents injury severity and the planned use of ≥2 plates reflects the anticipated extent of fixation based on preoperative surgical planning, these two distinct factors were retained in our predictive model. However, residual confounding may remain because more severe fractures are naturally more likely to require more extensive fixation. In addition, because this was a retrospective observational study, the identified factors should be interpreted as predictors associated with delayed wound healing rather than causal determinants.

Each variable in this scoring system was weighted according to its logistic regression coefficient to reflect its contribution to the overall risk. Although the presence of diabetes mellitus did not reach statistical significance in the multivariate analysis, it was included in the model because it has frequently been identified as a risk factor for impaired wound healing in previous studies [14-16]. The methodology used in this study aligns with that used to develop previously published models for predicting SSIs in orthopedic trauma [9] and nomograms for open tibial fractures [8], which also utilized preoperative variables and regression-based weighting. Unlike these general tools, our score is specific to closed distal fibular fractures, a common injury for which dedicated predictive models are lacking.

The primary strength and potential clinical implication of this scoring system lie in its simplicity and reliance on readily available clinical information, which may support early risk stratification and perioperative planning after further validation. This may facilitate a shift toward proactive, risk-adapted patient management. For instance, identifying high-risk individuals preoperatively may support informed patient counseling and consideration of tailored strategies, such as utilizing fibular intramedullary nails to minimize soft tissue disruption [20,21], using specific wound closure techniques, such as Allgöwer-Donati sutures [22], or implementing prophylactic negative pressure wound therapy [23]. This tool may help identify patients who could benefit from closer wound surveillance or preventive strategies, although its clinical utility should be confirmed in future validation studies.

This study had some limitations. First, it was a retrospective single-center study with a relatively limited cohort size, including only 30 delayed wound healing events, which may have introduced selection bias and increased the risk of model instability and overfitting despite bootstrap validation. Additionally, the model has not yet been externally validated, and its generalizability remains uncertain. Second, several factors that may influence wound healing, including surgical duration, timing of surgery, soft-tissue condition, vascular disease, nutritional status, HbA1c level, steroid or immunosuppressive use, antibiotic management, and wound closure methods, were not included because of the retrospective design, incomplete data availability, limited sample size, and the aim of developing a simple preoperative model. Third, although the planned use of ≥2 plates was generally determined preoperatively, the final number of plates may have been influenced by intraoperative findings. Finally, formal blinded assessment and interobserver reliability testing were not performed for fracture classification or wound assessment, which may have introduced assessment bias. Therefore, this scoring system should be considered preliminary until it is externally validated. Future prospective multicenter studies with standardized perioperative protocols and external validation cohorts are warranted to confirm the model’s clinical utility.

## Conclusions

In this study, we developed a simple preoperative risk scoring model to predict delayed wound healing after plate fixation in patients with closed distal fibular fractures using four clinically accessible variables: age, diabetes mellitus, fracture complexity, and planned use of ≥2 plates. The model demonstrated promising discriminatory performance and may help identify patients at higher risk of delayed wound healing before surgery. This scoring system may support preoperative risk stratification, patient counseling, surgical planning, and closer postoperative wound surveillance. However, because this was a single-center retrospective study with a relatively small sample size and internal validation only, the model should be regarded as preliminary. External validation in larger prospective multicenter cohorts is required to confirm its generalizability and clinical utility.
